# Comparative evaluation of lower respiratory tract microbiota in healthy and BRD-affected calves in Egypt

**DOI:** 10.1007/s11250-025-04322-w

**Published:** 2025-02-25

**Authors:** Ibrahim Sabry, Mohamed Zeineldin, Mohamed Kamal, Abdelghany Hefnawy, Hussam El-Attar, Yasein Abdelraof, Mohamed Ghanem

**Affiliations:** 1 Department of Animal Medicine, Faculty of Veterinary Medicine, Banha University, Banha, 13511 Egypt; 2 Department of Veterinary Hygiene and Management, Faculty of Veterinary Medicine, Cairo University, Cairo, 12211 Egypt

**Keywords:** Calves, Lung, Microbiome,16S rRNA gene, Respiratory disease

## Abstract

The advent of next-generation sequencing technologies has uncovered the importance of commensal microbial populations in the lower respiratory tract (LRT) for mucosal health and their role in the development of bovine respiratory disease (BRD). In this study, we aimed to characterize and compare the LRT microbiota in healthy and BRD-affected calves in Egypt. After assessing clinical respiratory scores in both groups, post-mortem lung samples from the cranial lobes of six clinically healthy calves and six calves affected by BRD were collected following slaughter. The most prevalent bacterial families in all samples were *Moraxellaceae* (11.06%), *Enterobacteriaceae* (8.23%), and *Flavobacteriaceae* (8.13%). The most common bacterial genera across all samples were *Acinetobacter* (13.1%), *Gracilibacillus* (7.9%), and *Pseudomonas* (5.0%). Notably, the overall microbial community structures differed significantly between healthy and BRD-affected calves. Alpha diversity analysis revealed significant differences in the Shannon (*p* = 0.0043) and Chao1 (*p* = 0.0001) indices between the two groups. This study highlights the substantial impact of BRD on the LRT microbiota of calves, highlighting the intricate relationship between host health and the LRT microbial ecosystem. Further investigations involving a larger sample size are necessary to establish the clinical significance of LRT microbiota in maintaining bovine respiratory health.

**Supplementary Information:**

The online version contains supplementary material available at 10.1007/s11250-025-04322-w.

## Introduction

Bovine respiratory disease (BRD), commonly known as shipping fever, poses a significant challenge to the health and economic well-being of cattle, particularly among recently weaned and transported calves (Taylor et al. 2010). Despite extensive efforts in disease prevention, BRD continues to be a leading cause of morbidity, mortality, welfare concerns, and financial losses in the cattle industry (Fulton 2009; Urban-Chmiel and Grooms 2012).

Recent studies have shed light on the critical role of the respiratory microbiota in maintaining respiratory health and providing "colonization resistance" against respiratory pathogens on the mucosal surface of the LRT (Lanaspa et al. 2017; Man et al. 2017). While traditional methods have been used to identify individual bacterial species and assess microbial composition, they are limited in providing direct molecular sequence information and are restricted to previously classified bacteria (Didelot et al. 2012). In contrast, high-throughput 16S rRNA marker gene sequencing has emerged as a commonly employed method for assessing microbial communities in cattle (Armougom and Raoult 2009; Zeineldin et al. 2019). Despite these advancements, as far as we are aware, there has been no research conducted on the variances in the bacterial microbiotas of the lower respiratory tract in healthy and BRD-affected calves in Egypt.

Given the crucial role of the respiratory microbiota in cattle health and disease, this study aimed to evaluate the difference in the LRT microbiota in both healthy and BRD-affected calves raised in Egypt. This study aimed to compare the LTR microbiota composition of BRD affected calves and healthy calves to evaluate how the microbial taxa vary between the two groups.

## Materials and methods

Post-slaughter cranial lung lobe tissue samples were collected from six feedlot calves (4–6 months old) diagnosed with Bovine Respiratory Disease (BRD) from two farms in Kafr Shukr, Kalyobyia, Egypt, following their slaughter for lung sample collection. The lung tissue samples were immediately frozen at -20 °C. The clinical health assessment of the calves involved in the study was carried out using the Wisconsin calf health scoring standards, which are a widely recognized tool for assessing the health status of cattle. These standards evaluate various parameters such as respiratory rate, cough, nasal discharge, ear position, eye condition, and rectal temperature. A respiratory score of 5 or higher, indicating the presence of at least two respiratory disease signs, classified a calf as having bovine respiratory disease (BRD) in this study. Additionally, six lung samples (cranial lobes) from clinically healthy calves, slaughtered at about 2.5 months of age, were collected from the Kafr Shukr slaughterhouse for comparison. Visual examination of the lungs was conducted, and tissue samples were then collected from the cranial lobes using sterilized forceps and scalpel blades. To prevent cross-contamination, disinfection procedures were performed between each sample collection. Forceps and scalpel blades were cleaned with a 20% sodium hypochlorite solution (Clorox®) for 3 min, followed by rinsing with sterile water and immersion in a 70% ethanol solution for 1 min before drying. The collected tissue samples were preserved at -20 °C.

For DNA extraction, the Power® Fecal DNA Isolation Kit from MO BIO Laboratories, Inc. (Carlsbad, CA, USA) was employed to extract DNA from tissue samples collected from both the BRD-affected and healthy calves. Subsequently, the integrity and quantity of DNA in each sample were assessed using a Nanodrop spectrophotometer by measuring the absorbance at 260 and 280 nm (NanoDrop Technologies, Rockland, DE, USA). The extracted DNA were stored at − 20 °C pending further analysis.

In this study, the Fluidigm Access Array system, in combination with the Roche High Fidelity Fast Start Kit, was employed to amplify the V1-V3 hypervariable region of the 16S rRNA gene (Welly et al. 2016). The primer sequences F28-2-for (ACACTGACGACATGGTTCTACA) and R519-2-rev (TACGGTAGCAGAGACTTGGTCT) were used to amplify the V1–V3 hypervariable region of the 16S rRNA gene, which was selected for its superior taxonomic resolution, as reported in several studies (Johnson et al. 2019). Following amplification, the DNA samples underwent quantification and were subsequently merged into equal pools. These pooled samples were then processed, and size selected using a 2 percent agarose E gel (Life Technologies, Grand Island, NY, USA). The cleaned and size-selected pooled products were further evaluated on an Agilent Bioanalyzer to ensure the correct profile and average size (data not shown). Subsequently, the final pooled Fluidigm libraries were submitted to the DNA Services lab at the Ministry of Defense for Illumina sequencing (San Diego, CA, USA). The Illumina MiSeq platform was utilized to sequence the V1-V3 region of the 16S rRNA gene, following Illumina's recommended practices. Sequencing was performed using paired-end reads with a read length of 2 × 250 bp using Illumina MiSeq v3.

The raw sequence data underwent preprocessing and analysis using Quantitative Insights into Microbial Ecology (QIIME®) version 1.9 (Caporaso et al. 2010). Chimeric sequences were identified and removed from the dataset using the UCHIME tool, following which the remaining sequences were taxonomically categorized up to the genus level by comparison to the Greengenes database (Edgar et al. 2011; McDonald et al. 2012). Operational taxonomic units (OTUs) were then assigned using the UCLUST method based on a 97 percent identity criterion, employing an open reference strategy (Prasad et al. 2015). Bacterial alpha diversity was assessed using the Chao1 and Shannon indices within the QIIME software, with random subsampling and rarefying to ensure a maximum of 1100 sequences per sample to account for variations in sequencing depth among different groups of calves. Beta diversity was visulaized through principal component analysis (PCA), with the prevalence of the most abundant bacterial genera as covariates (Bro and Smilde 2014).

Statistical analyses were conducted using JMP 13 software from SAS Institute Inc., North Carolina, USA. The median values of bacterial diversity indices and relative abundance of the LRT microbiota between healthy calves and calves with BRD were compared using non-parametric Wilcoxon/Kruskal–Wallis Tests fitted in JMP 13. Additionally, the UniFrac distances for the overall LRT microbiota between healthy calves and calves with BRD were assessed using nonparametric ANOSIM test (analysis of similarities) with 999 Monte Carlo permutations. Hierarchical clustering analysis of the LRT microbiota at the family, and genus levels was also performed to evaluate the variation between the two groups (Bridges 1966). A significance value of *p* < 0.05 was applied to all analyses.

## Results

The clinically healthy calves (*n* = 6) underwent thorough clinical examinations, revealing no observable injuries or diseases. These calves exhibited normal behavior during handling and examination, with vital indicators such as body temperature, respiration rate, and pulse rate falling within normal ranges at 39.42 ± 0.07°C, 48.28 ± 1.28 breaths per minute, and 101.96 ± 2.44 beats per minute, respectively. Furthermore, the mucous membranes of these calves appeared light rosy and red, indicative of their overall good health. In contrast, clinical examinations of the BRD-affected calves (*n* = 6) revealed a spectrum of clinical symptoms, including varying degrees of depression, shallow rapid respiration, loss of appetite, dyspnea, and the presence of purulent or mucopurulent nasal discharge as illustrated in (Figure S1**)**. Additional symptoms encompassed fever, redness of ocular mucous membranes with ocular discharge, cough, and in some cases, dry cough. The vital indicators of these calves, including body temperature, respiration rate, and pulse rate, were reflective of their condition, measuring at 40.5 ± 0.11°C, 58.22 ± 1.7 breaths per minute, and 140.66 ± 3.18 beats per minute, respectively.

The sequencing analysis of the V1-V3 hypervariable regions of the bacterial 16S rRNA gene produced a total of 1,230,307 reads across all lung samples, encompassing 1044 operational taxonomic units (97% identity cutoff). The dominant bacterial families across all samples included *Moraxellaceae* (11.06%), *Enterobacteriaceae* (8.23%), *Prevotellaceae* (6.62%), *Flavobacteriaceae* (8.13%) and *Bacillaceae* (6.55%) (Figure S2). At the genus level, the most predominant bacterial genera across all samples were *Acinetobacter* (13.1%), *Gracilibacillus* (7.9%), *Pseudomonas* (5.0%), and *Anaplasma* (3.7%) (Figure S3). Across all samples, there were significant inter-individual differences in the relative abundance of both family and genus levels (Figure S2 and Figure S3).

**Fig. 1:**
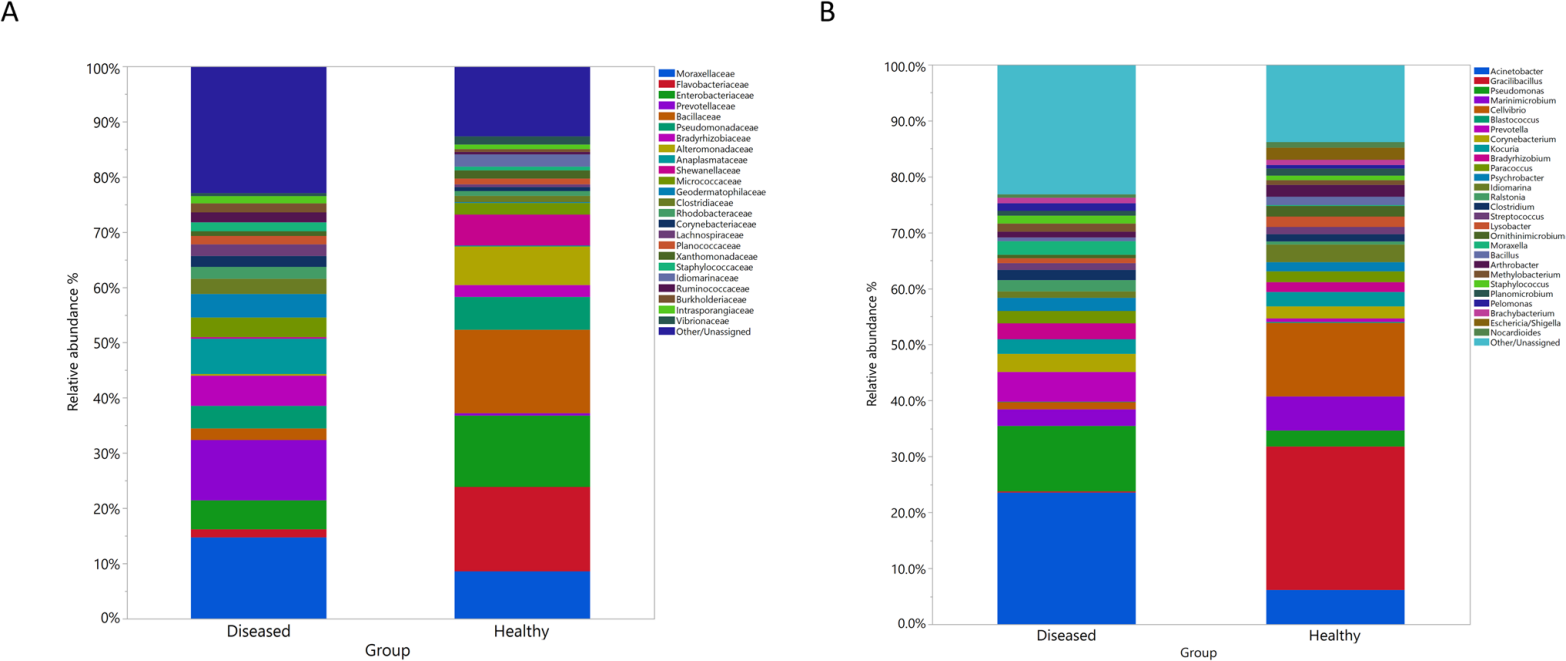
The relative abundance of top bacterial families (A) and bacterial genera (B) that averaged more than 1% of the relative abundance observed in the lung samples from BRD-affected and healthy calves

**Fig. 2:**
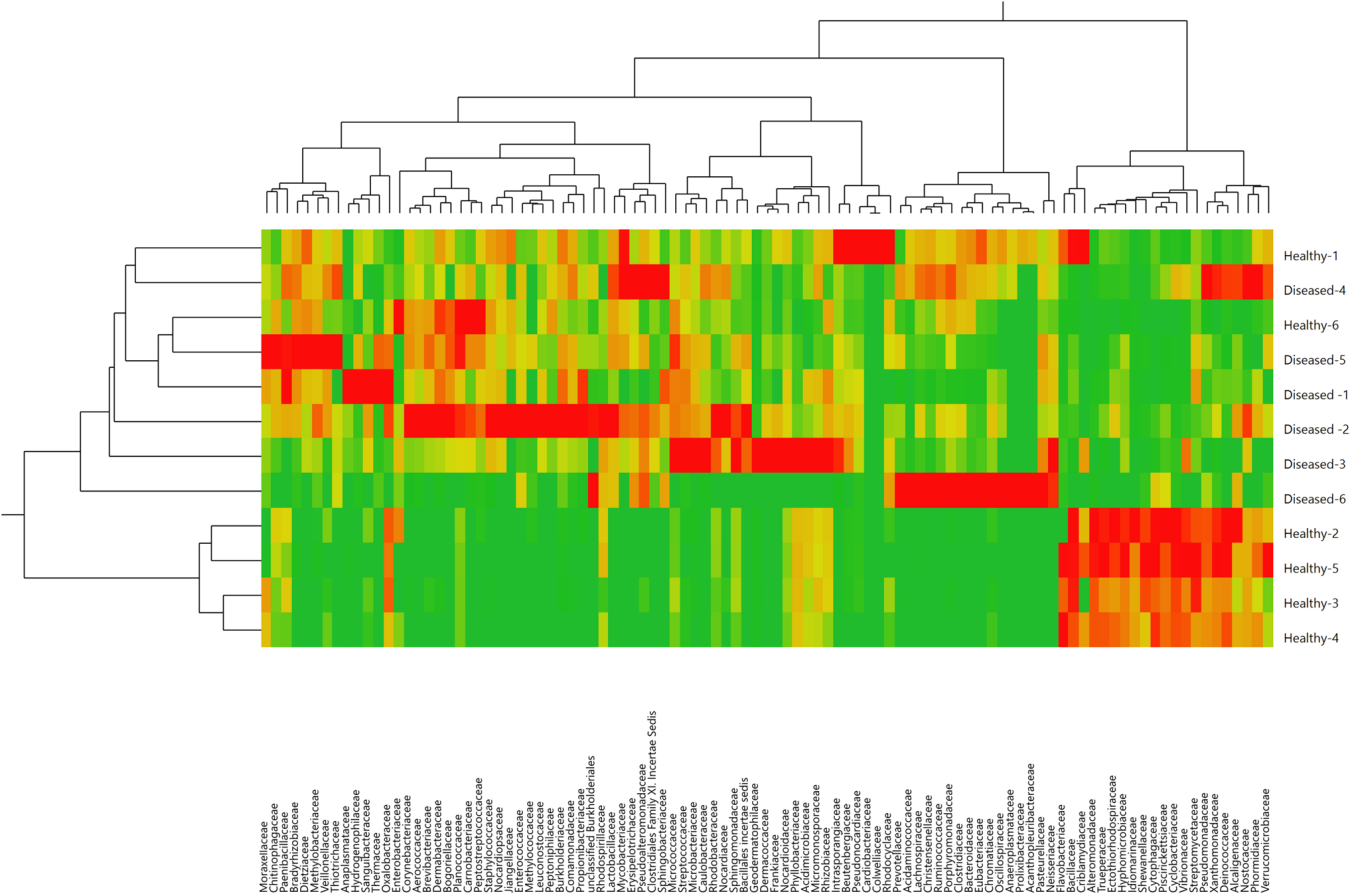
Hierarchical clustering analysis heatmap at the family level of the LRT microbiota among the healthy and BRD-affected calves

**Fig. 3:**
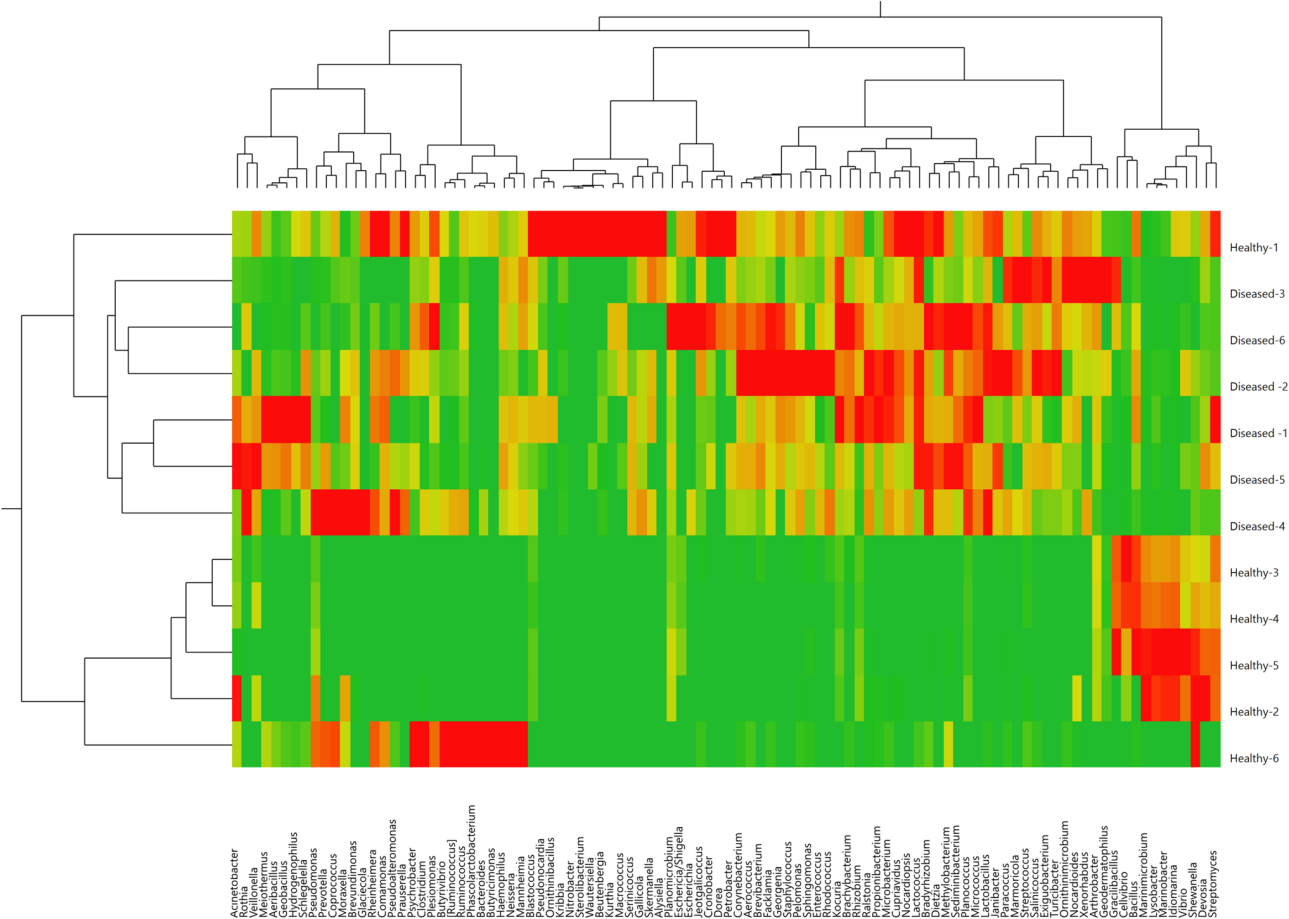
Hierarchical clustering analysis heatmap at the genus level of the LRT microbiota among the healthy and BRD-affected calves

At the family level, there were statistically significant variations between the two groups in the abundance of several bacterial families, including *Enterobacteriaceae* (*p* = 0.01), *Prevotellaceae* (*p* = 0.02), *Flavobacteriaceae* (*p* = 0.03), *Bacillaceae* (*p* = 0.04), *Anaplasmataceae* (*p* = 0.04), and *Geodermatophilaceae* (*p* = 0.04) (Fig. 1A**)**. At the genus level, there were statistically significant differences between the two groups in the abundance of several taxa, including *Gracilibacillus* (*p* = 0.04), *Pseudomonas* (*p *= 0.03), *Prevotella* (p = 0.04), *Aeromonas* (*p* = 0.04), *Escherichia/Shigella* (*p* = 0.02), *Euzebya* (p = 0.04), and *Aeribacillus* (*p* = 0.04) (Fig. 1B**)**. To further explore the relationships between bacterial communities in the two groups, we conducted a hierarchical clustering analysis using different bacterial taxa at the family (Fig. 2) and genus levels (Fig. 3). This analysis revealed clusters of taxa with high relative abundance present in both the healthy and BRD groups, providing insights into the dynamics of microbial community changes in calves.

**Fig. 4:**
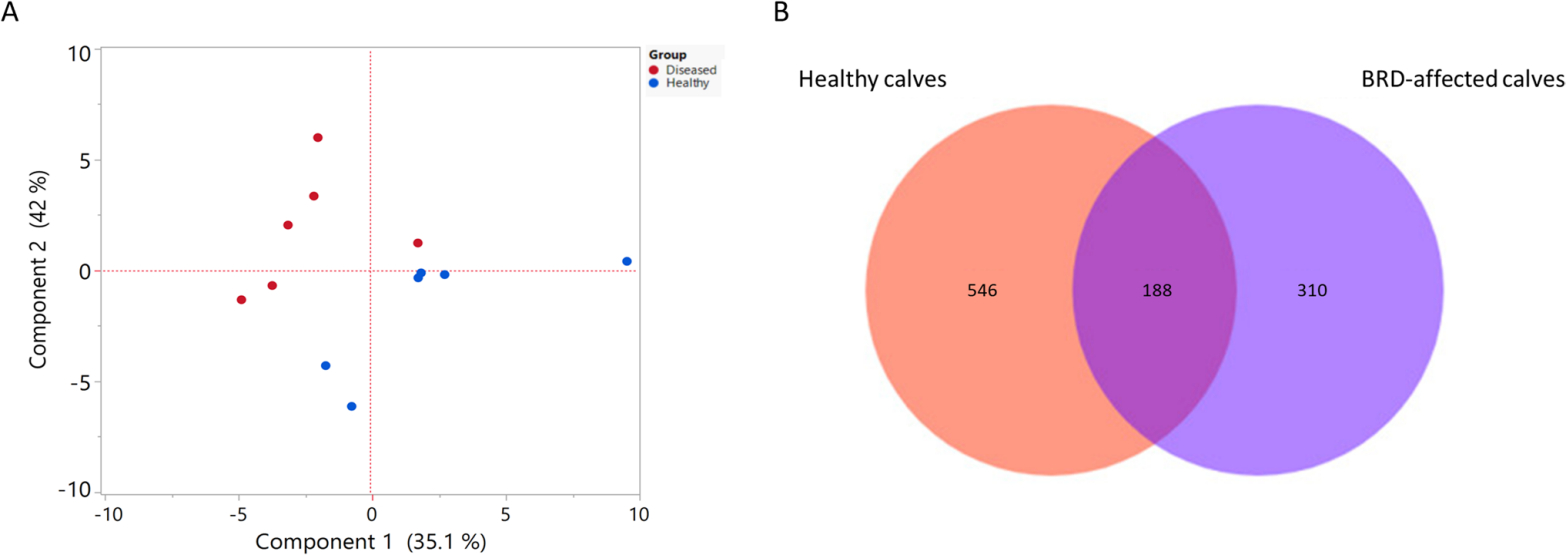
**A** Principal component analysis illustrating the relationship between the LRT microbiota in healthy and BRD-affected calves. **B** Venn diagram depicting the common and unique OTUs among the two groups (healthy and BRD-affected calves)

In assessing the overall variation in the LRT microbiota, we employed various metrics to measure alpha-diversity, including Chao1 and Shannon diversity index. Comparisons between healthy and BRD-affected calves revealed statistically significant differences in both Chao1 and Shannon diversity indices (Table 1), indicating distinct bacterial diversity between the two groups (*P* value < 0.05). Furthermore, to visulaize beta diversity, we used principal component analysis (PCA) to compare the healthy and BRD-affected communities. The results of the PCA, based on the most abundant bacterial genera, demonstrated a clear compositional distinction between the microbiota of healthy calves and those with BRD (ANOSIM R-value = 0.213, *P* value = 0.044) (Fig. 4A). Additionally, we generated a Venn diagram to visualize the distribution of OTUs and identify unique and shared OTUs between the two groups. The data set within the two groups comprised a total of 1044 OTUs, with 546 unique OTUs identified in healthy control calves and 310 unique OTUs in BRD-affected calves. Moreover, a core microbiota of 188 OTUs was found to be shared between the two groups, as illustrated in the Venn diagram (Fig. 4B).

**Table 1:** Bacterial diversity indices (Shannon and Chao1) measures for the LRT microbiota between the healthy and BRD-affected calves. There was a statistically significant difference in different bacterial diversity indices between the healthy calves and BRD-affected calves. *P* value < .05

Calves group	Healthy	BRD-affected	*P* value
Shannon index	5.22 ± 0.97	3.41 ± 0.71^*^	0.0043
Chao1 index	215.5 ± 36.42	116.33 ± 11.43^*^	0.0001

The values are presented as means ± standard error (Se)

## Discussion

Bovine respiratory disease (BRD) is a complex condition posing a significant challenge in cattle management, with implications for animal welfare and financial losses (Urban-Chmiel and Grooms 2012). Early detection and effective treatment of BRD are crucial in calves management, necessitating a deeper understanding of the underlying mechanisms and risk factors associated with the disease (Puig et al. 2022; Zeineldin et al. 2016; 2017a). The role of the LRT microbiota in promoting health and its contribution to the emergence of disease has drawn increasing attention, with mounting evidence suggesting its critical role in controlling the balance of local and systemic immune systems (Lanaspa et al. 2017). The study involved the examination of microbial communities in the LRT of healthy calves and those affected by BRD in the Egyptian feedlot settings.

The clinical examination of the calves diagnosed with BRD revealed a range of symptoms, including depression, shallow and rapid respiration, loss of appetite, dyspnea in some cases, and nasal discharge, which could be purulent or mucopurulent. These BRD-affected calves also exhibited elevated body temperature, respiratory rate, and pulse rate, aligning with findings from previous studies (Buczinski et al. 2014; Mahmoud et al. 2022; Zeineldin et al. 2016). While clinical examinations and rectal temperature measurements have traditionally served as prognostic indicators for assessing BRD severity, they may not provide a comprehensive understanding of lung tissue damage and functional alterations (Ramadan et al. 2019).

Recently, several research studies have recognized the mutually beneficial interactions between microbial populations and their host (Dale and Moran 2006; Zeineldin and Barakat 2023; Hooper et al. 2012; Maradiaga et al. 2014). Moreover, previous research has highlighted the pivotal role of respiratory microbiota composition in determining the health of the respiratory tract in cattle (Alexander et al. 2020; Zeineldin et al. 2017b, 2020). Our hypothesis suggests that disruptions in the LRT of calves could compromise their colonization resistance and significantly contribute to the development of BRD.

Upon examining the microbial composition in the LRT, we identified notable variations between healthy and BRD-infected cattle at the family and genus levels. These differences underscore the significance of microbial profiles in distinguishing between the two groups. The prevalent families *Moraxellaceae*, *Enterobacteriaceae*, and *Flavobacteriaceae* in the cattle's lower respiratory tract align with previous studies, emphasizing their importance in the cattle respiratory microbiome (Chai et al. 2022; Nicola et al. 2017). In line with other studies, we observed significant differences in their relative abundances between healthy and BRD-infected groups, indicating a disrupted microbiota in BRD-affected calves (Klima et al. 2019; Nicola et al. 2017). Notably, certain genera such as *Acinetobacter*, and *Pseudomonas* were markedly more prevalent in the lower respiratory tract of BRD-infected calves. These genera have been linked to opportunistic pathogens in respiratory infections across various animal species, suggesting a potential role in BRD pathogenesis or as indicators of dysbiosis in the lower respiratory tract (Qi et al. 2018; Xiao et al. 2021; Zeineldin et al. 2017c; Zhou et al. 2010).

A particularly interesting aspect of our study was the identification of high inter-individual differences in the relative abundance of LRT microbiota at both family and genus levels across all samples. This observation aligns with existing comprehensive research highlighting the multifaceted nature of factors influencing the composition of mucosal microbiota (Zeineldin and Barakat 2023; Zeineldin et al. 2017c). Previous studies have demonstrated that genetic, epigenetic, environmental, age, sex, and dietary influences collectively contribute to the establishment of mucosal microbiota, reflecting the intricate interplay of diverse determinants (Vercelli and Lynch 2023). It is important to recognize the significant genetic and epigenetic diversity present within the population, potentially contributing to the observed variations in the LRT microbiota. This emphasizes the complexity of the factors influencing microbial community composition, necessitating further exploration of the specific genetic and epigenetic influences that shape the distinctive microbial profiles observed in the study population (Man et al. 2017). Such investigations would provide valuable insights into the intricate relationships between host genetics, environmental elements, and the development of mucosal microbiota, ultimately contributing to a more comprehensive understanding of microbial dynamics in this context.

Analysis of alpha diversity unveiled significant differences in microbial richness and evenness between healthy and BRD-affected cattle, as evidenced by the Shannon and Chao 1 index. The reduced diversity observed in BRD-affected cattle implies a less resilient microbial community, associated with increased susceptibility to respiratory infections (Oliveira et al. 2020). Furthermore, beta diversity analysis, visualized by the PCA, distinctly delineated separate microbial communities between healthy and BRD-infected calves. This structural variation emphasizes the concept of dysbiosis in BRD cases, characterized by an imbalanced microbial community that may favor pathogenic species (Chai et al. 2022).

While the findings of this study are compelling, it is essential to acknowledge specific limitations. The analysis was conducted on a small number of samples due to the high cost of analysis, and further research with a larger cohort is needed to confirm these results. A major limitation is the collinearity between age and health status; age acts as a confounder, and due to this collinearity, we were unable to adjust for age in the data analysis. Consequently, it is difficult to determine if the observed differences in the microbiome are due to health status or age. Additionally, the bioinformatic processing and statistical techniques used, while common, have been shown to be suboptimal for microbiome studies. Advanced approaches, such as Amplicon Sequence Variants (ASVs) and compositionally aware statistical techniques, are now considered superior and should be considered in future studies. Moreover, the unavailability of negative controls and fragment analyzer data during sample analysis are a limitation. Finally, as the first study of its kind in Egypt, this research underscores the importance of understanding how environmental stressors impact the respiratory microbiota in Egyptian feedlot environments, warranting further investigation.

## Conclusion

In conclusion, this study shows the substantial impact of BRD on the lower respiratory microbiota in calves in Egypt. These findings highlight the differences in LRT between healthy and BRD-affected calves. Further research is required to develop targeted strategies for the management and prevention of BRD in calves, ultimately enhancing animal welfare and economic outcomes in the Egyptian cattle industry.

## Supplementary Information

Below is the link to the electronic supplementary material.

Supplementary file1 (DOCX 1503 KB)

## Data Availability

The corresponding author will make the data sets analyzed for the current work available based on request.
