# Supplementary material for: Comparative evaluation of lower respiratory tract microbiota in healthy and BRD-affected calves in Egypt
Source: Trop Anim Health Prod. 2025 Feb 25;57(2):78. doi: 10.1007/s11250-025-04322-w (PMC11861120; doi:10.1007/s11250-025-04322-w)

**Supplementary files:**

**Figure S1:** Clinical signs of BRD-affected calves showed serous and mucopurulent nasal discharge.


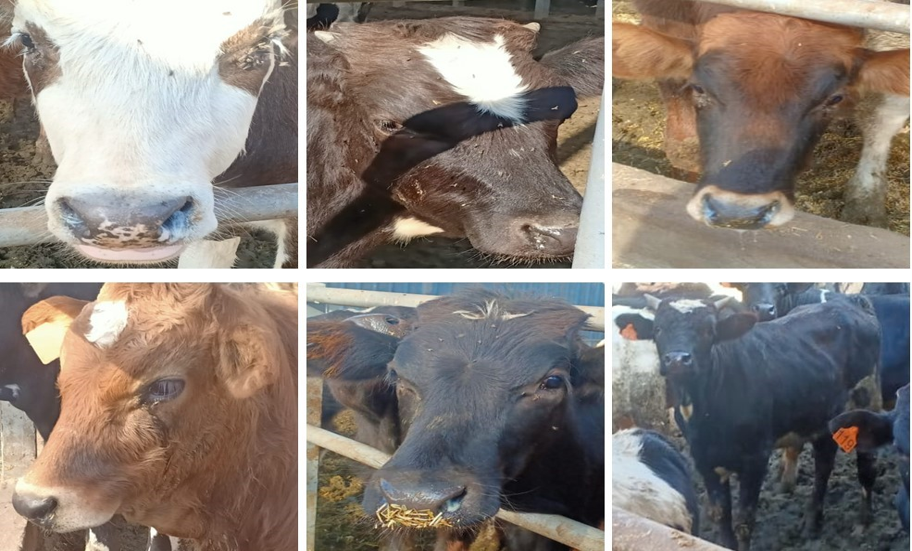


**Figure S2:** Relative abundance of the LRT microbiota at the family level detected in the cranial lobe lung samples across all samples.


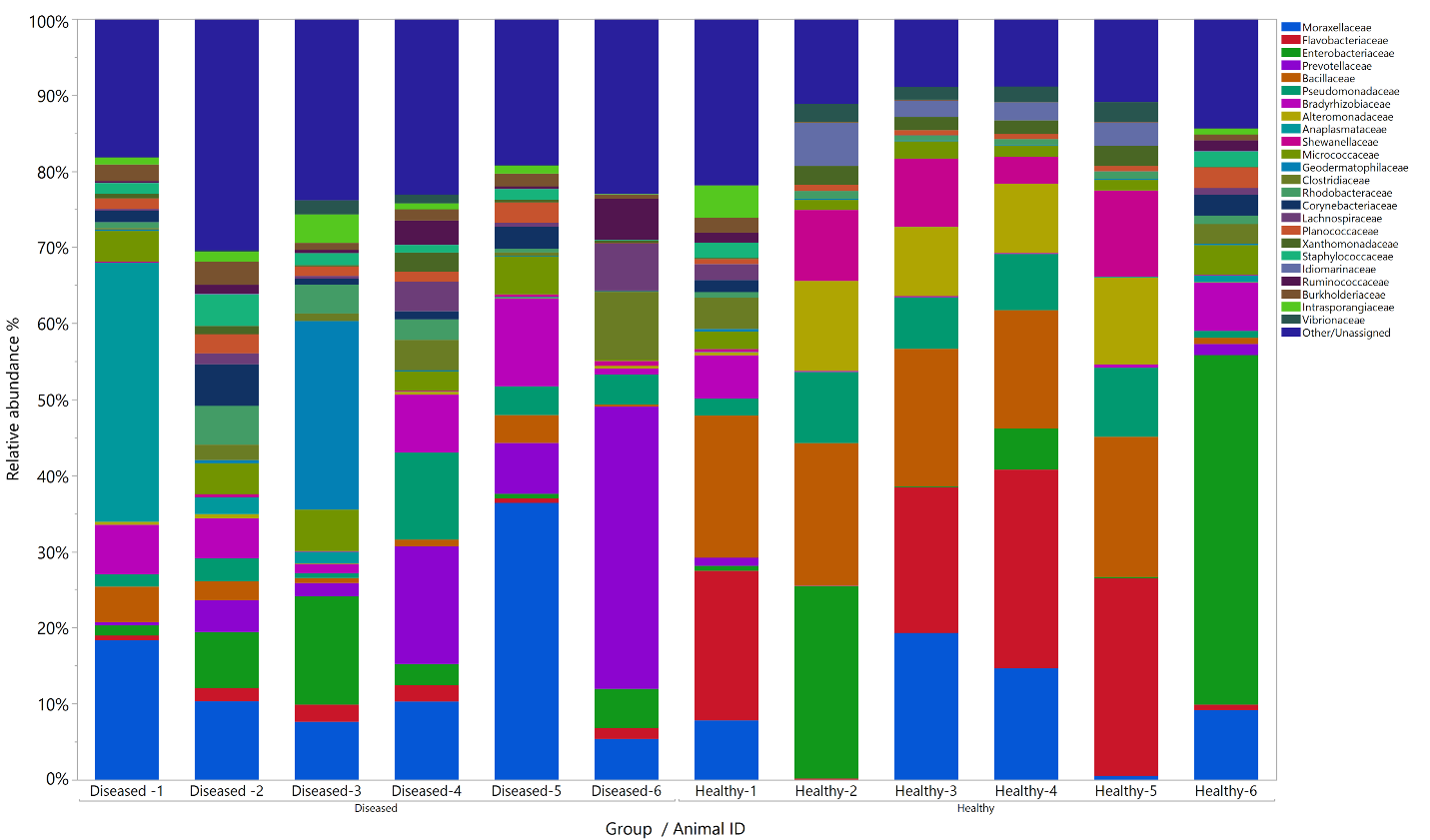


**Figure S3:** Relative abundance of the LRT microbiota at the genus level detected in the cranial lobe lung samples across all samples.


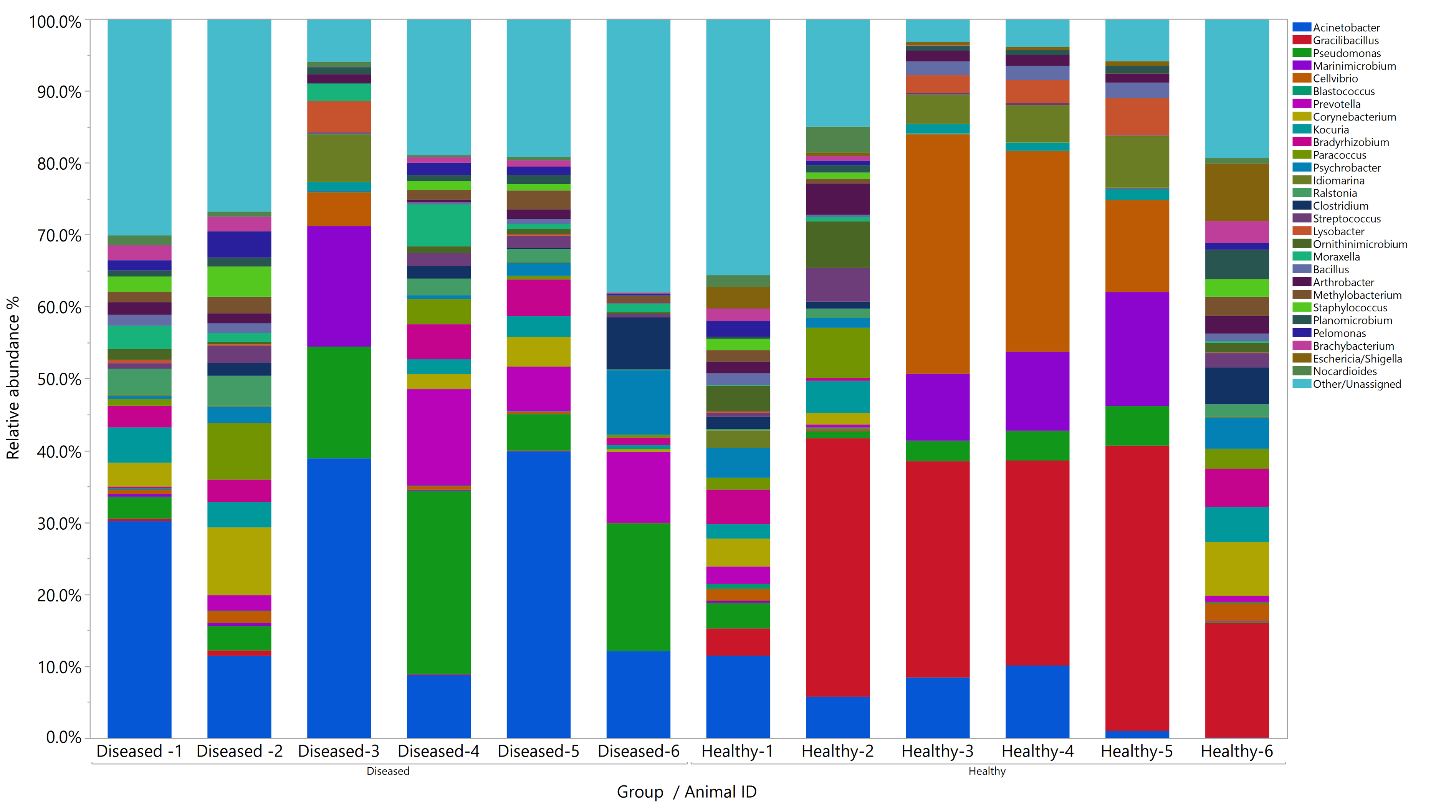

Supplement: Supplementary file 1 — Supplementary file1 (DOCX 1503 KB) [file 11250_2025_4322_MOESM1_ESM.docx]
